# LysR-Type Transcriptional Regulator VirR Responds to Temperature and pH and Directly Activates the Transcription of *virS*-Containing Operon in *Rhodococcus equi*

**DOI:** 10.1155/ijm/6618952

**Published:** 2025-01-03

**Authors:** Tsutomu Kakuda, Takashi Sato, Mari Takuhara, Hirofumi Hagiuda, Yasunori Suzuki

**Affiliations:** Laboratory of Animal Hygiene, School of Veterinary Medicine, Kitasato University, Higashi 23-35-1, Towada Aomori 034-8628, Japan

**Keywords:** *Rhodococcus equi*, VapA, VirR, VirS, virulence plasmid

## Abstract

*Rhodococcus equi*—a facultative intracellular pathogen of macrophages—causes bronchopneumonia in foals and patients who are immunocompromised. Virulent strains of *R. equi* possess a virulence-associated plasmid, which encodes a 15- to 17-kDa surface protein called virulence-associated protein A (VapA). VapA expression is regulated by temperature and pH. Two transcriptional regulators, VirR and VirS, are involved in the transcriptional regulation of *vapA*. VirR regulates VapA expression through VirS. However, whether VirR directly regulates *virS* transcription is unclear. In this study, we examined VirR binding to the promoter region of the *icgA* operon, which contains *virS*, using the electrophoretic mobility shift assay and DNase I footprinting. VirR bound DNA fragments containing the *virR*-*icgA* intergenic region. Transcription from the promoter in this region was VirR-dependent and regulated by temperature and pH. The VirR-binding site contained the LysR-type transcriptional regulator-binding consensus motif, T–N_11_–A. A point mutation (L98E) in the putative ligand-binding pocket of VirR constitutively activated the *icgA* promoter. However, no apparent difference was observed in the electrophoretic mobility shift assay and DNase I footprinting using the *icgA* promoter when L98E VirR was compared with wild-type VirR. A bacterial two-hybrid system identified an interaction between VirR and RpoA. Our data reveal that VirR binds the promoter of the *icgA* operon and directly activates its transcription. Furthermore, the regulation of VapA expression in response to temperature and pH is mediated by VirR.

## 1. Introduction

*Rhodococcus* is a gram-positive bacterium that belongs to the family *Nocardia* and is widely distributed in soil. Although > 60 *Rhodococcus* species are reported, only *Rhodococcus equi* infects animals and causes disease [1]. *R. equi* infects horse, cattle, goat, llama, camel, alpaca, pig, boar, dog, cat, etc. [2–11]. *R. equi* causes severe, fatal respiratory disease in young horses < 6 months of age and is pathogenic to humans, causing pneumonia in immunocompromised individuals [12]. *R. equi* possesses a virulence plasmid, and plasmid-cured *R. equi* strains were avirulent in a foal or mouse infection model [13, 14]. *R. equi* is thought to have expanded the niche into various animal species by acquiring this virulence plasmid.

The virulence plasmid has a pathogenicity island (PAI) that is thought to be acquired by horizontal transmission; PAI contains multifamily genes that encode virulence-associated proteins (Vaps) [15]. Of these, *vapA* encodes a 15- to 17-kDa cell surface protein. The deletion of *vapA* reduces pathogenicity to the level of plasmid curing [13, 16–18]. *R. equi* can survive and multiply in host macrophages [19]. Intracellular growth depends on VapA, which affects the membrane permeability of *R. equi*–containing phagosomes, excluding the proton-pumping ATPase, and consequently, neutralizing the phagosome lumen [20, 21]. VapA expression is regulated by the temperature and pH of the bacterial environment. VapA expression is abolished at normal temperature (≤ 30°C) and increases at higher temperature and lower pH (e.g., 37°C and pH 6.5), which *R. equi* encounters in the early endosomes of macrophages [22, 23].

The transcriptional regulation of *vapA* involves two transcriptional regulators, VirR and VirS, which are encoded in the same PAI as *vapA* [24–26]. VirS is a response regulator of a two-component regulatory system and belongs to the OmpR/PhoB subfamily, although its sensor kinase is unknown [15]. The deletion of *virS* markedly reduces *vapA* promoter activity [24]. The transcription of *virS* is regulated by temperature and pH, and the *vapA* promoter is constitutively activated when VirS is constantly expressed from an exogenous promoter [24]. *virS* forms an operon with three upstream genes, including *icgA*, *vapH*, and *orf7*. The transcriptional regulation of this operon involves VirR, which is a lysR-type transcriptional regulator (LTTR) [26]. Mutations in *virR* markedly reduce the transcription of this operon [24].

LTTRs are the largest regulatory family in prokaryotes and are involved in regulating biological activities, such as metabolism, quorum sensing, oxidative stress response, motility, and virulence [27]. LTTRs require low molecular weight ligands, referred to as coinducers, to activate gene expression [27, 28]. Although most LTTR family members bind their target promoters in both the absence and presence of the coinducer, interactions with coinducers elicit structural changes in the LTTR–promoter complex, resulting in transcriptional activation [27].

In this study, we identified the VirR-binding site upstream of the *icgA* operon promoter and characterized it by electrophoretic mobility shift assay (EMSA) and DNase I footprinting. We used site-directed mutagenesis to substitute VirR residues in a region that corresponds to the ligand-binding sites of other LTTRs. L98E VirR allowed activation of the *icgA* operon promoter under noninducing conditions. These results suggest that VirR directly regulates the transcription of the *icgA* operon by binding upstream of the promoter as a positive regulator, responding to temperature and pH, probably by binding an unidentified coinducer.

## 2. Materials and Methods

### 2.1. Bacterial Strains and Growth Conditions

*R. equi* ATCC33701 was cultured on brain heart infusion (BHI) agar at 30°C. Apramycin (80 *μ*g/mL) was added to BHI agar to select for *R. equi* growth. All *R. equi* strains were stored at −80°C in 85% BHI broth and 15% glycerol (vol/vol). *Escherichia coli* DH5*α* and ER2566 were cultured on Luria–Bertani (LB) agar or in LB broth. Ampicillin (50 *μ*g/mL) was used when necessary. *E. coli* strains were stored at −80°C in 85% LB broth and 15% glycerol (vol/vol).  Table S1 lists the strains used in this study.

### 2.2. Construction of Transcriptional Reporter Using *t*-Tag

The *virR*–*icgA* intergenic region (−116 to +4), fused with *t*-tag [25] (P_*icgA*_2-*t*-tag), located within two Rv3813 terminators of *Mycobacterium tuberculosis* [29], and flanked by the *Not*I site, was synthesized and cloned into pUC57-mini (pTKR918). The fragment of P_*icgA*_2-*t*-tag flanked by the Rv3813 terminator was excised from pTKR918 with *Not*I and ligated into the *Not*I site of pINT to create pReporter. The ATCC33701 strain or *virR*_Δ*HTH*_ strain (TKR474) was transformed with pReporter by electroporation and cultured on BHI agar with apramycin for transformant selection. The nucleotide deletion or substitution derivatives of pReporter were generated using PCR amplification with the pReporter template, and the following primer pairs are as follows: delta-35-1-delta-35-2 and delta ABS1-delta ABS2, RL1 and RL2 (Table S2), and KOD One PCR Master Mix. The PCR mix was *Dpn*I digested for enrichment of PCR-amplified plasmids. DH5*α* was transformed by PCR-amplified plasmid and selected on LB agar containing ampicillin. Deletion or substitution was confirmed by sequencing. TKR474 was transformed with the resultant plasmids by electroporation.

### 2.3. Construction of Complement Plasmid

The fragment containing the *virR* ORF and its own promoter region was amplified by PCR using the P_*virR*_-termF–virR-R primer pair (Table S2) and ligated into the pGEM-T Easy Vector to create pTKR509. To introduce point mutations in the *virR* coding sequence, PCR-mediated mutagenesis was performed using the complementary mutagenic primer pair VirR L98E-1–VirR L98E-2 (Table S2), pTKR509 template, and KOD One PCR Master Mix. The PCR mix was *Dpn*I digested to enrich PCR-amplified plasmids. DH5*α* was transformed by PCR-amplified plasmid and selected on LB agar containing ampicillin. The point mutation was confirmed by sequencing. To create the integration plasmid containing P_*icgA*_2-*t*-tag with P_*virR*_-*virR* (wild) or P_*virR*_-*virR* (L98E), pTKR509 and pTKR917 were used as templates to amplify fragments containing P_*virR*_-*virR* (wild or L98E) by using the virR-builderF–virR-builderR primer pair (Table S2), respectively. The resultant PCR products and EcoRV-digested pReporter were assembled by NEBuilder, according to the manufacturer's instructions, to create pTKR913 and pTKR914, respectively. The virulence plasmid-cured derivative of ATCC33701 was transformed with pTKR913 or pTKR914.

### 2.4. RNA Extraction

Total bacterial RNA was isolated from 5-mL cultures grown to midlogarithmic phase (optical density at 600 nm [OD_600_] = 0.8). Briefly, cultures were mixed immediately with 10 mL of RNAprotect Bacteria Reagent (Qiagen) and incubated for 5 min at 20°C–28°C. Cells were harvested by centrifugation for 10 min at 5000 × g at 4°C, resuspended in 0.7 mL of RLT buffer (RNeasy Mini Kit; Qiagen), mixed with 1 g of 0.1-mm-diameter zirconia–silica beads (*μ*T-01, Taitec), and lysed by mixing three times for 1 min in a bead beater (Taitec) at 4600 rpm. Total RNA was isolated using RNeasy Mini Kit (Qiagen), according to the manufacturer's instructions. To remove contaminant DNA, RNA was treated with 10 U of RNase-free DNase for 30 min at 37°C. DNase was inactivated by incubating the mixture for 5 min at 75°C.

### 2.5. qRT-PCR

In total, 200-ng RNA was reverse transcribed to cDNA using 6-mer oligonucleotide primers and a PrimeScript RT-PCR kit (TaKaRa), according to the manufacturer's instructions. When the expression of *t*-tag was examined, cDNA was reverse transcribed using t-tag 167 R primer. Real-time RT-PCR analysis was performed in 20 *μ*L, containing 1 × PowerSYBR Green PCR Master Mix (Applied Biosystems), 200 nM forward and reverse primers, and cDNA. The primers used are listed in Table S2. Reactions were performed with StepOne Real-Time PCR Systems (Applied Biosystems) as follows: 95°C for 10 min, 40 cycles at 95°C for 15 s, and 60°C for 1 min. Melting curve analysis was performed after each reaction to ensure the specificity of PCR products. Primer efficiency was calculated using a standard curve derived from dilutions of plasmid containing each target gene. Primer pairs with amplification efficiency ranging from 90% to 110% were used. Relative quantification was performed using standard curves generated for each gene-specific primer pair for all experiments, except one where results were normalized to *gyrB* levels, and analyzed using the ΔΔCT method. The values shown are relative to *gyrB* values.

### 2.6. 5′-RACE

To determine transcriptional initiation site of the *icgA* operon, 5′-RACE was performed with 5′-Full RACE Core Kit (TaKaRa), according to the manufacturer's instructions. First-strand cDNA was synthesized from 3 *μ*g of total RNA using 5′-terminal phosphorylated RT primer (Table S2). The cDNA was treated with RNase H to degrade RNA from hybrid DNA–RNA. The single-stranded cDNA was cyclized (or concatenated) with T4 RNA ligase. PCR was performed with the cyclized (or concatenated) cDNA with the A1–S1 primer pair, followed by nested PCR using the A2–S2 primer pair (Table S2). The amplified DNA fragments were ligated into pGEM-T Easy Vector.

### 2.7. Plasmid Integration in *virR*_ΔHTH_ Strain

PCR-mediated mutagenesis was used to introduce point mutations into the coding sequence of *virR* in pTKR528 (pINT::P_*virR*_-*virR*). pTKR638 (pINT::P_*virR*_-*virR* L98E), pTKR639 (pINT::P_*virR*_-*virR* S100E), and pTKR640 (pINT::P_*virR*_-*virR* L101E) were produced using the primer pairs VirR L98E-1-VirR L98E-2, virR A100E-1-virR A100E-2, and virR L101E-1-virR L101E-2, respectively (Table S2). Each plasmid was electroporated into TKR474 (virR_*ΔHTH*_, P_*vapA*_-*lacZ*). Transformants were recovered on BHI agar containing 80 *μ*g/mL apramycin.

### 2.8. *β*-Galactosidase Assay

Cells were cultured overnight at 30°C in BHI broth with shaking. Cultures were diluted 1:10 with 60 mM Tris-buffered BHI (pH adjusted to 6.5 or 8.0). Cultures were grown to OD_600_ = 0.5–0.7. Cells were washed twice with 0.9% NaCl, resuspended in 500 *μ*L of *Z* buffer (60 mM Na_2_HPO_4_, 40 mM NaH_2_PO_4_, 10 mM KCl, 1 mM MgSO_4_, and 50 mM *β*-mercaptoethanol [pH 7.6]), permeabilized with 20 *μ*L chloroform and 35 *μ*L of 0.1% SDS, and incubated at 28°C for 5 min with 100 *μ*L of 13 mM 2-nitrophenyl *β*-D-galactopyranoside (Sigma-Aldrich, St Louis, MO, USA). The reaction was stopped by adding 250 *μ*L of 1 M Na_2_CO_3_, and absorbance was read at 420 nm using a spectrophotometer (GENESYS 20, Thermo Fisher Scientific). The activity of each sample was calculated in Miller units as follows: 1000 × OD_420_/OD_600_ × reaction time × volume. Data represent average results of three biological replicates.

### 2.9. Protein Expression and Purification

DNA extracted from *R. equi* strain ATCC33701 was used as template. A DNA fragment containing the *virR* ORF was amplified by PCR using the VirR-NdeF–VirR-SapR primer pair. The PCR product was electrophoresed on 0.8% agarose gel. The gene fragment was gel extracted using MinElute kit (Qiagen), digested with *Nde*I and *Sap*I, and ligated into *Nde*I and *Sap*I sites of pTXB1 (New England Biolabs) to construct pTKR752. *E. coli* strain ER2566 was transformed with pTKR752. Bacteria were cultured in 200-mL LB broth with ampicillin to midexponential phase, incubated with 0.4 mM isopropyl-D-thiogalactopyranoside (IPTG) overnight at 18°C, harvested, disrupted by sonication, and centrifuged at 20,000 × g for 30 min at 4°C. The supernatant was loaded onto a chitin column (New England Biolabs) and equilibrated with column buffer (20 mM Tris-HCl [pH 8.0] and 500 mM NaCl). The column was first washed with column buffer, then quickly washed with cleavage buffer (column buffer + 50 mM DTT), and incubated overnight at 20°C. VirR was eluted with column buffer. Recombinant protein was dialyzed against PBS for 12 h and stored at −30°C until use. PCR-mediated mutagenesis was used to introduce point mutations into the coding sequence of *virR* in pTKR752 by using the VirR L98E-1–VirR L98E-2 primer pair to produce pTKR901. pTKR901 was electroporated into ER2566. L98E VirR was produced as described previously.

### 2.10. EMSA

The *virR*–*icgA* intergenic region was amplified by PCR using the P_*icgA*_-F–P_*icgA*_-R primer pair. The amplified DNA fragment was cloned into pGEM-T Easy Vector to construct pTKR837, which was used as template to construct the mutated versions of P_*icgA*_ by PCR-based mutagenesis using the following primer pairs: delta-ABS1–delta-ABS2, delta-L1–delta-L2, delta-R1–delta-R2, and RL1–RL2 (Table S2). The binding of VirR to the *icgA* operon promoter was investigated using the EMSA. Probes containing the *virR*–*icgA* intergenic region were obtained by PCR using pTKR837, pTKR778, pTKR780, or pTKR781 as template. The reverse primer (P_*icgA*_-R) was FITC-labeled at the 5' terminus (Table S2). The amplified fragments were separated by agarose gel electrophoresis, and the probes were extracted from the gel by MinElute kit. The reactions (15 *μ*L) for measuring mobility shift contained FITC-labeled DNA probes and different concentrations of VirR in a buffer containing 10 mM Tris-HCl (pH 7.5), 100 mM KCl, and 1 mM EDTA, 5% vol/vol glycerol, 0.1 mM DTT, and 10 μg/mL BSA. Reactions were performed at room temperature for 30 min, loaded onto 6% polyacrylamide/bis (37.5:1) gels in 0.5 × Tris–borate–EDTA buffer, and run at a constant voltage of 150 V for 90 min. Gel images were acquired using a Typhoon scanner (GE Healthcare) and analyzed using ImageQuant software (GE Healthcare).

### 2.11. DNase I Footprinting Assays

Probes were obtained by PCR using a combination of FITC-labeled and FITC-unlabeled primers (Table S2). Binding reactions were performed in DNase I footprinting buffer (20 mM Tris-HCl, 3 mM MgCl_2_, 5 mM CaCl_2_, 100 mM NaCl, 100 mM EDTA, 50 μg/mL BSA, and 0.1 mM DTT) containing 100-ng probe and 0–0.975 μM VirR in a final volume of 100 μL. The reaction was incubated at 37°C for 30 min and maintained at 25°C for 5 min. Partial digestion of DNA was initiated by adding 1 μL of DNase I stock solution (50 μg/mL). Incubation was continued for 1 min, and the reaction was stopped by adding 100 μL of phenol–chloroform and 25 μL of stop solution (1.5 M sodium acetate, 20 mM EDTA, and 10 mg/mL tRNA). The sample was centrifuged, and the supernatant was ethanol precipitated. DNA was resuspended in 5 μL of loading buffer (0.125% bromophenol blue, 0.125% xylene cyanol, 20 mM EDTA, and 95% formamide) and separated by gel electrophoresis on a 6% polyacrylamide–urea sequencing gel. A sequencing reaction performed with the Sequenase 2.0 kit (USB), using an oligonucleotide specific for the labeled strand, was run in parallel as a size marker. Images of the gels were acquired using a Typhoon scanner (GE Healthcare) and analyzed using ImageQuant software.

### 2.12. Circular Permutation Analysis

Circular permutation analysis was performed to assess DNA bending by VirR using the pBend2 vector, as described [30]. The oligonucleotides PicgA-core1 and PicgA-core2 were annealed, and the resultant double-stranded DNA was ligated into *Sal*I and *Bam*HI sites of pBend2 to construct pTKR810. Plasmid pTKR810 was single digested with *Eco*RV and *Bam*HI. Removal of restriction enzyme and buffer change were performed using a PCR purification kit (Qiagen). The binding reaction was performed using 0.75 *μ*M VirR and 100 ng of each digest as probe. Assay conditions were identical to those for EMSA. Samples were separated on a 6% polyacrylamide native gel in 0.5 × Tris–borate–EDTA buffer at 4°C. Gels were stained with ethidium bromide, and DNA bands were visualized by UV fluorescence. The bending angle (*α*) was calculated using the Thompson and Landy [31] equation *μ*_*M*_/*μ*_*E*_ = cos (*α*/2), where *μ*_*M*_ = mobility of the complex with protein bound at the center of DNA and *μ*_*E*_ = mobility of the complex with protein bound at the end.

### 2.13. Bacterial Two-Hybrid System

A bacterial two-hybrid system was used to identify interactions between VirR and RpoA. This system is based on functional complementation between two fragments (T18 and T25) of the catalytic domain of adenylate cyclase from *Bordetella pertussis* [32]. The plasmids pT18 and pT25 express proteins fused to the N-terminus of T18 and C-terminus of T25, respectively. *virR* (wild), *virR* (L98E), and *rpoA* were PCR amplified using the following primer pairs: T18-VirRF–T18-VirRR, T25-VirRF–T25-VirRR, T18-RpoAF–T18-RpoAR, and T25-RpoAF–T25-RpoAR (Table S2) and assembled into the MCS of pT25 and pT18 using NEBuilder, according to the manufacturer's instructions. The insertions were confirmed by sequencing. *Escherichia coli* strain Sp850 (*cya*) was transformed with all combinations of pT18 and pT25 plasmids containing *virR* (wild), *virR* (L98E), or *rpoA*. The negative control contained Sp850 with pT18 and pT25, and the positive control was Sp850 with pT18-Zip and pT25-Zip [32]. Transformants were cultured in LB broth at 30°C with 100 μg/mL ampicillin, 30 μg/mL chloramphenicol, and 0.5 mM IPTG. *β*-galactosidase assays were done in triplicate; data represent average results of three biological replicate experiments.

## 3. Results

### 3.1. The *icgA* Operon Is Transcribed From a Promoter in the *virR*–*icgA* Intergenic Region

**Figure 1:**
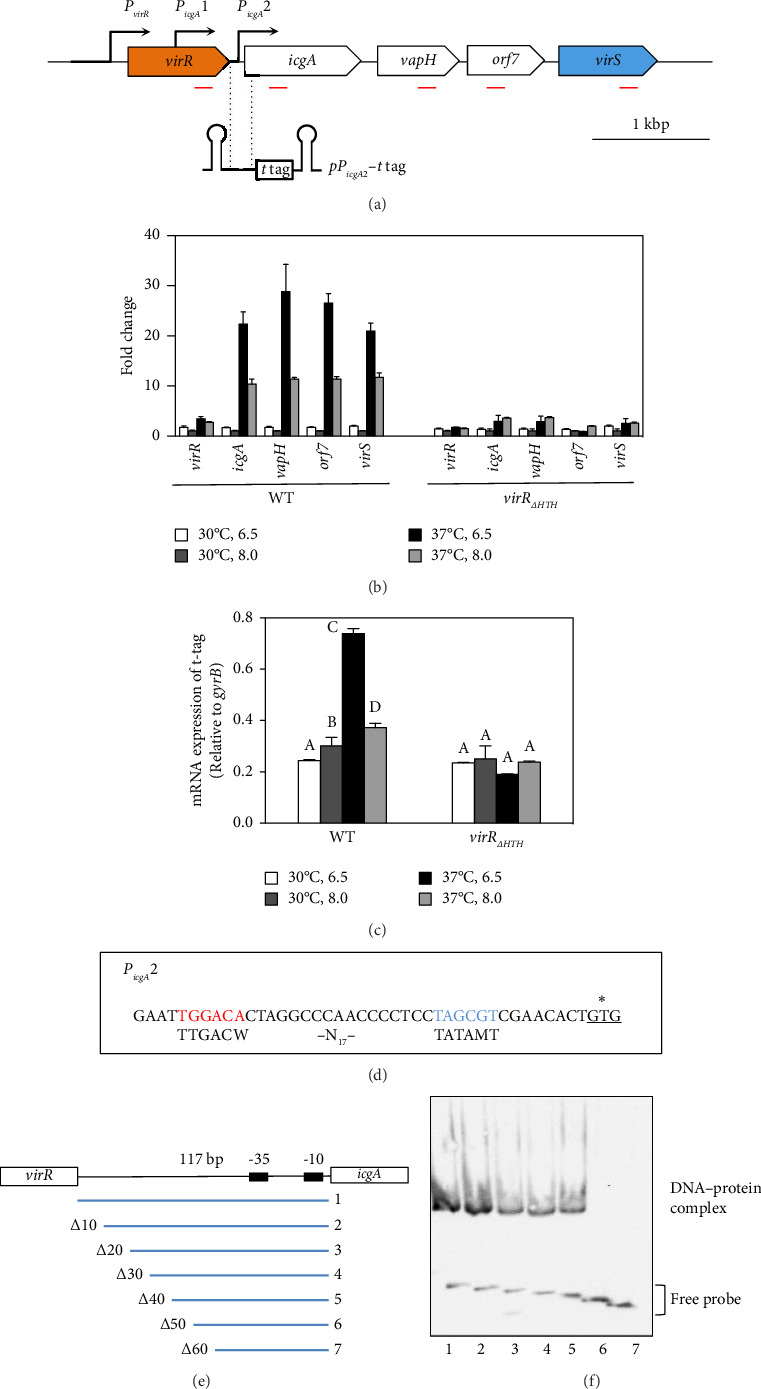
Transcriptional regulation of the *icgA* operon. (a) Map of the region extending from *virR* to *virS*. The red lines below the map indicate the positions of amplicons of each gene examined in part *B*. Schematic representation of the insert in pTKR830. The hairpin structure represents transcriptional terminators. (b) Transcriptional regulation of the *icgA* operon by temperature and pH. mRNAs were isolated from ATCC33701 under the conditions indicated and analyzed using real-time RT-PCR. Data were normalized to the levels of *gyrB* and analyzed using the ΔΔCT method. Values represent increase in the amounts of mRNA relative to that expressed by bacteria cultured at 30°C, pH 8.0. Data are shown as mean ± SEM of three independent experiments with two technical replicates each. (c) Transcriptional regulation of P_*icgA*_2. pTKR830 was introduced into wild-type or *virR*_Δ*HTH*_ strain. mRNAs were isolated from each strain cultured under the conditions indicated and analyzed using semiquantitative real-time RT-PCR. Values shown are relative to *gyrB* levels. Data represent average results from three technical replicates. Experiments were repeated at least three times with similar results. Error bars indicate standard deviations. (d) The promoter sequence of the *icgA* operon determined in this study. The consensus promoter sequence of *Mycobacterium tuberculosis* SigA is indicated below the sequence. An asterisk shows a transcription initiation site. The start codon of *icgA* is underlined. (e) The probes used in part *F* were shortened by 10 bp from the 5′ end. (f) Electrophoretic mobility shift assay (EMSA) for VirR binding to the *virR*–*icgA* intergenic region. The EMSA reactions (15 μL) for measuring mobility shift contained the FITC-labeled DNA probe and VirR. Lane 1, full length; Lane 2, −10 bp; Lane 3, −20 bp; Lane 4, −30 bp; Lane 5, −40 bp; Lane 6, −50 bp; Lane 7, −60 bp.

*virS*, which regulates *vapA* transcription, constitutes an operon with *icgA*, *vapH*, and *orf7*. The transcription of this operon is regulated by temperature and pH [24]. We confirmed the mRNA level of the four genes constituting the *icgA* operon (*icgA*, *vapH*, *orf7*, and *virS*) by real-time RT-PCR. The expression of *icgA*, *vapH*, *orf7*, and *virS* remarkably increased (> 21- to 29-fold) when *R. equi* was cultured at pH 6.5°C and 37°C compared with that at pH 8.0°C and 30°C (Figure 1(a)). The *icgA* operon was reportedly transcribed from a promoter within an ORF of *virR* upstream of *icgA* (P_*icgA*_1, Figure 1(b)) [25]. We evaluated the mRNA level using a pair of primers that amplifies the downstream region of P_*icgA*_1 within *virR*. We found that the level of mRNA containing this region increased, but the change was ∼3-fold. Because the transcriptional changes of these regions are different (7- to 10-fold), we postulate that the *icgA* operon is transcribed from a promoter other than P_*icgA*_1. To examine this hypothesis, we constructed an *R. equi* strain that contains the *virR*–*icgA* intergenic region, followed by PCR-detectable *t*-tag in the chromosome (Figure 1(a)), and examined the level of transcription by real-time RT-PCR. The maximum expression of *t*-tag was observed when bacteria were cultured at 37°C and pH 6.5 and transcription was significantly reduced at pH 8.0 (Figure 1(c)), suggesting that the transcription of the *icgA* operon occurred from and was controlled by the *virR*–*icgA* intergenic region. We performed 5'-RACE to determine the transcription initiation site of the *icgA* operon. We found that the transcription initiation site was 1 bp upstream from the start codon of *icgA*. Sequences similar to TATAMT (−10) and TTGACW (−35), which are binding motifs for *M. tuberculosis* SigA [33], were found upstream of the transcription initiation site (TAGCGT and TGGACA, respectively) (Figure 1(d)). We designated this promoter P_*icgA*_2.

### 3.2. VirR Binds to the *virR-icgA* Intergenic Region

VirR has a positive effect on the transcription of the *icgA* operon [24], but whether this effect is directly mediated by VirR binding to the *icgA* promoter is unclear. We used EMSA to examine the binding between DNA upstream of *icgA* and VirR. When recombinant VirR was added, we detected a band shift (Figure 1(f)). When the probes were shortened by 10 bp from the 5' end (Figure 1(e)), the shifted band disappeared in the probe shortened by 50 bp (Figure 1(f)). These results suggest that VirR binds to the *virR*–*icgA* intergenic region and that the binding region is located within 77 bp upstream of *icgA*.

### 3.3. The L98E Mutation in VirR Leads to a Constitutive Active State of the *vapA* Promoter

**Figure 2:**
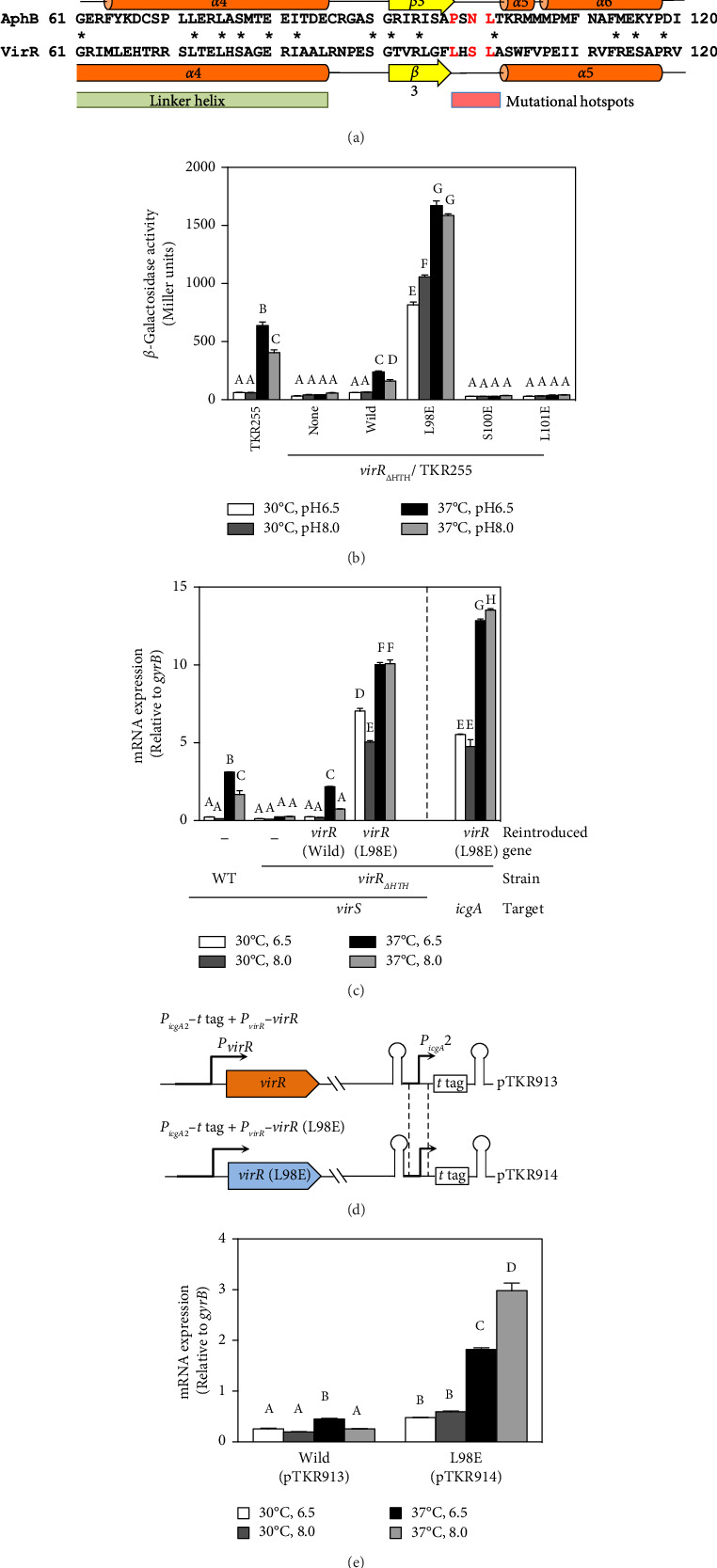
Mutational analysis of the putative ligand-binding region of VirR. (a) Linear protein alignments of AphB and VirR. Only the N-termini are shown. Secondary structure domains of AphB and VirR, based on the crystal structure of AphB [30], are indicated above and below the sequence, respectively. Residues targeted for mutagenesis are indicated by red letters. Proteins were aligned with GENETYX-MAC. Identical residues are indicated by an asterisk. (b) Influence of substitutions in the ligand-binding region (L98E, S100E, and L101E) on the expression of the *vapA* promoter in *Rhodococcus equi* at pH 6.5 or 8.0°C and 30°C or 37°C. The P*vapA-lacZ* (TKR255) and *virR*_Δ*HTH*_ strains of TKR255 (TKR474) were used. The integration vector (pINT) containing wild-type, L98E, S100E, and L101E *virR* with its own promoter was introduced into TKR474. *β*-Galactosidase activity of the indicated strains was determined in Miller units. Error bars represent standard deviations for the results of three biological replicates. (c) Transcription levels of *icgA* and *virS* in the VirR (L98E)-expressing strain. mRNAs were isolated under the conditions indicated and analyzed using real-time RT-PCR. Values shown are relative to *gyrB* levels. Data represent average results from three technical replicates. Experiments were repeated at least three times with similar results. Error bars indicate standard deviations. (d) Schematic representation of pTKR913 and pTKR914. (e) pTKR913 or pTKR914 was introduced into the virulence plasmid-cured derivative of the ATCC33701 strain. mRNAs were isolated from each strain cultured under the conditions indicated and analyzed using semiquantitative real-time RT-PCR. Values shown are relative to *gyrB* levels. Data represent average results from three technical replicates. Experiments were repeated at least three times with similar results. Error bars indicate standard deviations.

The N-terminal region (∼80 residues) of LTTR is relatively conserved across the LTTR family [27]. This region contains a helix-turn-helix domain (involved in DNA binding) and a linker helix (involved in protein intermolecular binding) [27]. The amino acid sequence of VirR did not show high similarity with that of *Vibrio cholerae* AphB, whose crystal structure is known [34]; however, a comparison of their secondary structures showed high similarity in the domain from the helix-turn-helix domain to *α*6 of AphB (Figure 2(a)). In LTTR family members, the region between *β*3 and *α*5 contains amino acids that form part of the coinducer-binding pocket involved in coinducer binding [27]. Because this region contains mutational hotspots, coinducer binding and responsiveness are altered [35–41]. P98, N100, and L101 mutations in AphB allow it to activate the promoter under nonpermissive culture conditions [34]. It is unclear if VirR requires coinducer binding for activation. To examine whether mutations in this region affect VirR function, we performed site-directed mutagenesis at positions corresponding to amino acid residues that lead to the formation of a constitutively active AphB. Wild-type and mutant VirR were expressed in a loss-of-function mutant of *virR*, and the activity of the *vapA* promoter was measured by the *β*-galactosidase reporter assay. High activity of the *vapA* promoter was observed in the strain expressing VirR with the L98E mutation even under noninducible conditions (30°C) (Figure 2(b)). The other mutations abolished the activity of the *vapA* promoter. To examine if the L98E mutation of VirR affects the transcription of the *icgA* operon, we determined the transcriptional levels of *icgA* and *virS.* The transcription of both genes markedly increased even under nonpermissive culture conditions when L98E VirR was expressed (Figure 2(c)). To further examine if P_*icgA*_2 is involved in this transcriptional control, P_*icgA*_2-*t*-tag was introduced in the virulence plasmid-cured strain with wild or mutant *virR* (Figure 2(d)). The transcription of t-tag significantly increased when L98E VirR was expressed (Figure 2(e)). These results suggest that the L98E mutation renders VirR constitutively active and could activate P_*icgA*_2 even under nonpermissive culture conditions.

### 3.4. Wild-Type and L98E VirR Bind to the *icgA* Promoter in the Same Manner

**Figure 3:**
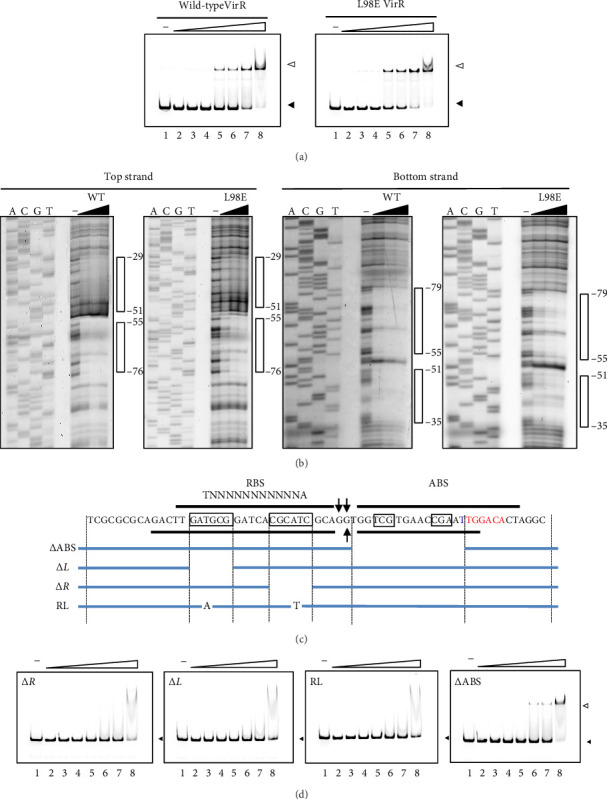
(a) EMSA of wild-type VirR and L98E VirR to the *icgA* operon promoter. Protein concentrations (μM) were 0 (Lane 1), 0.15 (Lane 2), 0.24 (Lane 3), 0.30 (Lane 4), 0.48 (Lane 5), 0.60 (Lane 6), 0.74 (Lane 7), and 0.97 (Lane 8). Closed and open arrowheads indicate free probe and the DNA–protein complex, respectively. (b) DNase I footprinting of wild-type VirR and L98E VirR at the *icgA* operon promoter region. FITC-labeled probes were incubated with various amounts of protein. Triangles above the figures represent increasing amounts of each protein (0, 0.24, 0.48, and 0.75 μM). Open bars on the side represent regions protected by wild-type VirR and L98E VirR and are labeled relative to the transcription initiation site. DNA ladders of the corresponding promoter region are shown on the left. (c) Summary of wild-type and L98E VirR binding in the DNase I footprinting assay. Sequence of the *icgA* operon promoter region is shown. Bars above (top strand) and below (bottom strand) the sequence indicate DNase I protection. Boxes indicate partial dyad symmetry. Arrows denote hypersensitive positions. The −35 box is indicated by a red letter. RBS and ABS are recognition and activation binding sites, respectively (see Discussion section). The location of the lysR-type transcriptional regulator (LTTR)–binding site (LTTR box) is shown by T–N_11_–A. The locations of deletions (ΔABS, Δ*L*, and Δ*R*) and substitution (RL) in the probe used in *D* are shown. (d) EMSA of wild-type VirR to mutated version of the *icgA* operon promoter. Protein concentrations (μM) were 0 (Lane 1), 0.15 (Lane 2), 0.24 (Lane 3), 0.30 (Lane 4), 0.48 (Lane 5), 0.60 (Lane 6), 0.74 (Lane 7), and 0.97 (Lane 8). Closed and open arrowheads indicate free probe and DNA–protein complex, respectively.

Because the *vapA* promoter was constitutively transcribed in the strain expressing L98E VirR, L98E VirR activates the *icgA* promoter even under noninducible conditions, leading to increased VirS levels and *vapA* promoter activity. If VirR binds to the coinducer and changes to the active form, the L98E mutation may mimic the conformational change of VirR due to coinducer binding. In other LTTRs, complexes of genes and bound transcription factors show different mobilities in EMSA from genes bound to transcription factor without coinducer [42]. To examine this hypothesis, EMSA was performed using wild-type VirR and L98E VirR. No difference was observed in complex mobility between mutant and wild-type VirR (Figure 3(a)). We performed DNase I footprinting to examine the binding site of wild-type and L98E VirR in detail. Wild-type VirR protected two regions of the top strand (−76 to −55 and −51 to −29) relative to the transcriptional start site of the *icgA* operon (Figure 3(b)). The downstream region (−51 to −29) was more weakly protected than the upstream region (−76 to −55). Two hypersensitive sites (−53 and −54) were observed between these protected regions, where the intensity of the band increased. In the bottom strand, two regions (−79 to −55 and −51 to −35) were protected, with a hypersensitive site in between. Similar protected regions and hypersensitive sites were observed with L98E VirR. Thus, wild-type and L98E VirR bind identical sites in the upstream region of P*icgA*2.

### 3.5. Mutations in the LTTR-Binding Site Abolish VirR Binding

In the upstream region out of the two regions protected in DNase I footprinting, we identified a 17-bp region of partial dyad symmetry from position −74 to −58 in the top strand containing T–N_11_–A, which is characteristic of the LTTR-binding site (LTTR box) (Figure 3(c)). To examine whether this region is involved in VirR binding, gene fragments lacking the left (Δ*L*) or right (Δ*R*) half of the LTTR box or bearing substitutions from T–N_11_–A to A–N_11_–T (RL) were subjected to EMSA. Complex formation with VirR was not observed in all the cases tested (Figure 3(d)). These results suggest that this region is necessary for VirR binding. A decrease of free band and formation of a V-shaped complex were observed when the concentration of VirR was > 0.97 *μ*M (also observed in Lane 8 of Figure 1(a), right). However, this binding was observed even with unrelated DNA fragments at this concentration (data not shown). Therefore, it was considered nonspecific binding.

### 3.6. The Downstream Binding Region Is Required for Maximum VirR Binding and Promoter Activation

**Figure 4:**
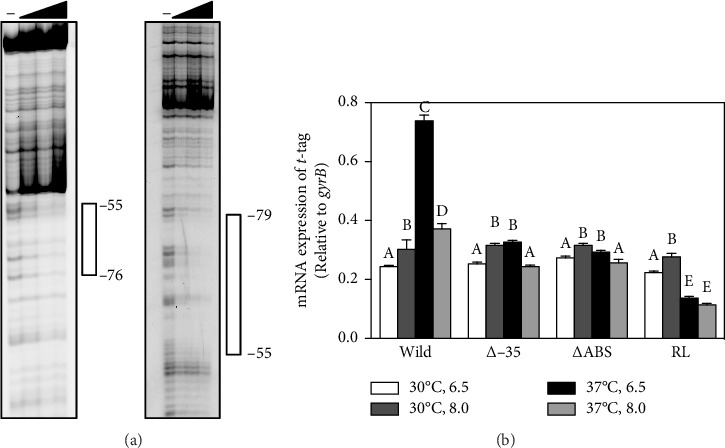
(a) DNase I footprinting of wild-type VirR on the ΔABS probe. FITC-labeled probes were incubated with various amounts of protein. The triangles above the figures represent increasing amount of protein (0, 0.24, 0.48, and 0.75 μM). The open bars on the side of the figures represent regions protected by wild-type VirR and are labeled relative to the transcription initiation site. (b) Transcriptional level of mutated P_*icgA*_2. pTKR830 (wild), pTKR919 (Δ−35), pTKR826 (ΔABS), and pTKR943 (RL) were introduced into the wild-type strain. mRNAs were isolated from each strain cultured under the conditions indicated and analyzed using semiquantitative real-time RT-PCR. Values shown are relative to *gyrB* levels. Data represent average results from three technical replicates. Experiments were repeated at least three times with similar results. Error bars indicate standard deviations.

Although the region from −51 to −29 was protected by VirR, no LTTR box was found within this region. To examine the effect of this region on VirR binding, gene fragments lacking the region from −51 to −34 were subjected to EMSA. Complex formation was concentration-dependent; however, compared with the wild-type fragments (Figure 3(a)), complex formation required a higher concentration of VirR (Figure 3(d)). These results suggest that the sequences within this region have a small but significant effect on VirR binding. Next, we tested the effect of deleting this region by DNase I footprinting. Regions −76 to −55 and −79 to −55 were protected in the top and bottom strands, respectively, but the downstream region was unprotected (Figure 4(a)). This result suggests that VirR specifically binds to the downstream region, although no LTTR box is included. To examine the effect of the downstream region in VirR-mediated transcriptional activation, this region was deleted from P_*icgA*_2-*t*-tag and introduced into the wild-type strain. The transcriptional level of t-tag reduced to that of the −35 deletion and RL mutation (Figures 3(c) and 4(b)) when the downstream region was deleted (Figure 4(b)), suggesting that VirR binding to this region is necessary for P_*icgA*_2 activation.

### 3.7. The Bending Angle of DNA by Wild-Type VirR and L98E VirR Is Approximately 35°

**Figure 5:**
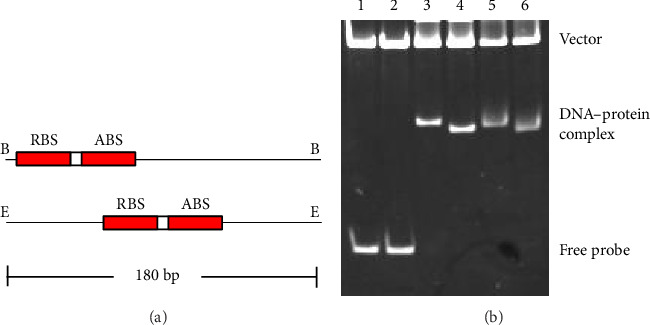
Circular permutation analysis. (a) The position of the VirR-binding site in the fragments when pTKR810 was digested with *Bam*HI (B) or *Eco*RV (E). (b) Circular permutation analysis of wild-type VirR and L98E VirR binding to the *icgA* operon promoter region. The probe pBEND2, containing the VirR-binding region of the *icgA* operon promoter, was digested with *Eco*RV (Lanes 1, 3, and 5) and *Bam*HI (Lanes 2, 4, and 6). Wild-type VirR (Lanes 3 and 4) and L98E VirR (Lanes 5 and 6) are added into the reactions at a final concentration of 0.75 μM.

The presence of hypersensitive sites between the two VirR-protected regions suggests that VirR bends the *icgA* promoter upon binding. In other LTTRs, changes in the bending angle in the promoter influence transcriptional regulation [27, 43]. To examine if VirR bends DNA, a standard circular permutation assay was performed. A 50-bp gene fragment containing the protected region was cloned into the *Xba*I and *Sal*I sites of pBend2. When this plasmid is cleaved with *Eco*RV and *Bam*HI, respectively, DNA fragments of the same size are excised from the vector. When the plasmid is cleaved with *Eco*RV or *Bam*HI, the VirR-binding region is at the center or end of the excised DNA, respectively (Figure 5(a)). The DNA fragment with the highest or lowest mobility is expected to bind VirR at the end or middle, respectively. The bending angle is estimated from the relative electrophoretic mobility retardation caused by bending. The complex with wild-type VirR ran faster when *Bam*HI was used (Figure 5(b)). The bending angle caused by wild-type VirR was estimated to be 35°. The mobility of the complex upon L98E VirR binding was similar to that upon wild-type VirR binding.

### 3.8. VirR Interacts Each Other and Binds to RpoA

**Figure 6:**
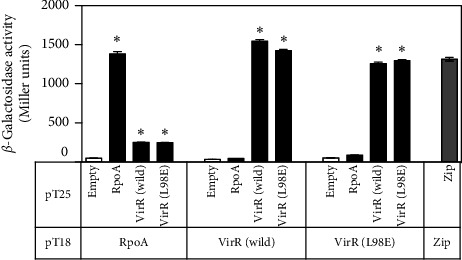
*β*-Galactosidase assays of bacterial two-hybrid system interactions. When fused to proteins that interact, fragments T25 and T18 of adenylate cyclase associate with activated cyclic AMP (cAMP) synthesis, resulting in the transcription of *lacZ*. T25 and T18 were fused to a leucine zipper (zip) for positive control [32]. Data represent average results from three biological replicates. Error bars indicate standard deviations.

Most LTTRs bind DNA by forming homotetramers [44]. A bacterial two-hybrid system was used to analyze the interactions between VirR proteins [32]. In *E. coli*, where VirR was expressed as a fusion protein with T18 and T25, *β*-galactosidase activity was found to be similar to that of the positive control, the leucine zipper region of the yeast protein GCN4, which was also expressed as a fusion protein with T18 and T25 (Figure 6). This suggests that VirR proteins bind to each other with high affinity. VirR L98E had comparable *β*-galactosidase activity. When RpoA-T18 and T25-VirR were expressed, *β*-galactosidase activity was significantly higher than when T18 and T25 were expressed. Similar levels of *β*-galactosidase were detected in RpoA-T18 and T25-VirR L98E, suggesting that wild-type and L98E interact similarly with RpoA.

## 4. Discussion

VapA expression is regulated by temperature and pH [22, 23]. Two transcriptional regulators, VirS and VirR, are involved in this regulation [24–26]. The transcription of *vapA* is regulated by VirS, whose expression is transcriptionally regulated by VirR [24]. Additionally, *virS* transcription is regulated by temperature and pH, and the expression of VirS resulted in VapA expression in the absence of VirR even under noninducible conditions [24]. Therefore, quantitative changes in VirS may regulate VapA expression through these environmental stimuli. We found that P_*vapA*_ was constitutively active in the strain that expressed mutant VirR (L98E). This result suggests that VirR is responsive to temperature and pH. A substitution at this residue renders VirR constitutive active as a transcriptional regulator.

Many LTTRs bind to a small molecule called the coinducer, which leads to structural changes, thereby controlling transcription [27]. It is unclear whether the coinducer is required for VirR-mediated transcriptional regulation, but in other LTTRs, amino acid residues in the region subjected to site-directed mutagenesis in this study are involved in coinducer binding [28, 45, 46]. Mutations in this region led to a coinducer-independent phenotype in many LTTRs [35–41]. Therefore, the coinducer may be involved in the regulatory function of VirR. Its cellular levels may change in response to temperature and pH, and by binding to VirR, it may change the amount of VirR that has undergone a structural change and regulate *virS* transcription.

The *icgA* operon is transcribed from the *virR* promoter (P_*virR*_) and P_*icgA*_1 that is located within the coding region of VirR [25, 26]. P_*icgA*_1 is responsible for the transcriptional regulation of the *icgA* operon by temperature and/or pH [25]. However, this study indicated that P_*icgA*_2, between *virR* and *icgA*, is involved in VirR-mediated transcriptional regulation. Furthermore, 5'-RACE identified promoter sequences similar to the promoter consensus sequences of SigA and HrdB in *M. tuberculosis* and *Streptomyces coelicolor*, respectively. The *virR* and *vapA* promoters reportedly have similar sequences to the promoter consensus sequences of HrdB [25, 26]. SigA and HrdB are the principal sigma factors in each species, which are major components of the sigma factor during exponential cell growth. The features of their promoters indicate that VapA expression mainly occurs during the exponential growth phase [47]. IcgA is a transport protein belonging to the major facilitator superfamily. Deletion of *icgA* enhanced the bacterial growth within macrophages and significantly reduced macrophage viability [48]. The transcript of P_*icgA*_2 starts 1 bp upstream from the IcgA-coding ORF. Full-length IcgA is unlikely to be translated from this transcript because it lacks a ribosome binding sequence. Therefore, IcgA may be translated from mRNA transcribed from P_*virR*_ and P_*icgA*_1, because transcripts spanning *virR* and *icgA* were detected by an operon mapping experiment [25].

The results of EMSA and DNase I footprinting showed that VirR binds to the upstream region of P_*icgA*_2. Deletion of the HTH of VirR markedly reduced *virS* transcription [24]. This study showed temperature and pH regulate transcription from P_*icgA*_2, which is abolished in the *virR*_Δ*HTH*_ mutant (Figure 1(c)). Taken together, we conclude that VirR is the direct activator of P_*icgA*_2. DNase I footprinting showed VirR protected a wide region of ∼43 bp, which was divided into two regions interrupted by a hypersensitive site. An interrupted palindrome was found in the upstream protected region, which contained the T–N_11_–A motif, called the LTTR box [27]. No palindrome or LTTR box was found in the downstream protected region. Destruction of the palindrome and substitution of T–N_11_–A in the upstream protected region abolished VirR binding, although the downstream binding region was intact. Conversely, when the downstream protected region was deleted, VirR binding was concentration-dependent, but complex formation required a higher concentration of VirR, compared with the wild-type fragments (Figures 3(a), 3(d)). In the DNase I footprinting assay, the mutant probe displayed protection of the upstream region, whereas the downstream region was unprotected (Figure 4(a)). Transcription from this promoter was abolished when the downstream region was deleted (Figure 4(b)). These features in the nucleotide sequence of the binding site and the binding property of the transcriptional regulator are consistent with reports on other LTTRs [38, 49–51]. Therefore, we believe that the upstream and downstream regions correspond to the recognition and activation binding sites, respectively.

In the bacterial two-hybrid experiment, high *β*-galactosidase activity was detected when VirR-T18 and T25-VirR were coexpressed, suggesting that VirR monomers interact. Consistently, other LTTRs were reported to form homomers. With a few exceptions, such as CrgA and BenM, LTTR generally forms a complex with DNA as a homotetramer. LTTRs form a protomer through the linker helix domain, which connects the N-terminal helix-turn-helix domain with the C-terminal cofactor-binding domain. In addition, the protomers bind each other through their interface and form a tetramer (dimer of dimer). The high *β*-galactosidase activity of VirR-T18- and T25-VirR-coexpressed bacteria is presumed to reflect the stability of the coiled-coil structure formed between the linker helix domains of VirR. Considering the protected region in DNase I footprinting, VirR may form a tetramer and a complex with DNA, like other LTTRs.

In other LTTRs, conformational changes in the complex between regulator and DNA caused by the coinducer–regulator binding are detected as changes in mobility in EMSA and protected regions in DNase I footprinting. In AtzR of *Pseudomonas* sp. strain ADP, the presence of the coinducer, cyanuric acid, increased mobility in EMSA and decreased the protected region in DNase I footprinting [38]. Inducer-dependent shortening of the protected region in DNase I footprinting and relaxation of regulator-induced DNA bending are important features of the model proposed for LTTR activation, the so-called sliding dimer model [42, 52–56]. In this study, if the L98E substitution in VirR mimics structural changes due to coinducer binding, some differences in them might be observed. However, we did not detect differences in mobility and protected region between wild-type and mutant VirR. The reason for the discrepancy between in vivo and in vitro experiments is unclear. Crystal structure analysis of *Klebsiella aerogenes* CisB showed that the sulfate anion, and not the original coinducer, *N*-acetylserine, binds to the coinducer-binding site and is retained even after protein purification and crystallization [28, 57]. The ion-bound structure is believed to mimic the coinducer-bound active conformation. Consistently, in VirR, ions or small molecules might have bound VirR during the expression and/or purification of recombinant protein, resulting in the formation of an active conformation of wild-type VirR. In circular permutation analysis, no difference was found in the estimated bending angle between wild-type VirR and mutant VirR. The bending angle of DNA bound to wild-type VirR is ∼35°, which is considerably smaller than the bending angle of DNA in the absence of coinducer, reported in other LTTRs (range: 50°–100°) [27]. These results suggest that wild-type VirR is in an active conformation.

Another possible explanation for this discrepancy is that mutant VirR activates P_*icgA*_2 by a mechanism other than the sliding dimer model. Some LTTR family members become activated by coinducer binding, which changes their binding properties with RNA polymerase and promoter DNA [58]. Although DNA binding by wild-type VirR and L98E was similar, they may differ in their ability to attract RNA polymerase to the promoter and stabilize its DNA binding. In this study, RpoA binding was similar between wild-type and L98E. Studies must investigate their binding properties with other subunits of RNA polymerase, such as sigma factor.

In conclusion, this study revealed that VirR binds to the *icgA* operon promoter as a positive transcriptional regulator. The transcriptional response of the *icgA* operon to temperature and pH is mediated by VirR, although the mechanism of transcriptional activation by VirR is unclear and warrants further investigation.

## Data Availability

The data that support the findings of this study are available from the corresponding author upon reasonable request.

## Supporting Information

Supporting Information 1 Table S1: The bacterial strains used in this study are listed in Table S1.

Supporting Information 2 Table S2: The primers and oligonucleotides used in this study are listed in Table S2. Their nucleotide sequences and purposes are shown.
