# Supplementary material for: LysR-Type Transcriptional Regulator VirR Responds to Temperature and pH and Directly Activates the Transcription of virS-Containing Operon in Rhodococcus equi
Source: Int J Microbiol. 2025 Jan 3;2025:6618952. doi: 10.1155/ijm/6618952 (PMC11724031; doi:10.1155/ijm/6618952)
Supplement: Supporting Information 1 — Table S1: The bacterial strains used in this study are listed in Table S1. [file 6618952.f1.docx]

| Table S1. Bacteria and plasmids used in this study | | | |
| --- | --- | --- | --- |
| Bacterial species  and plasmids | Bacterial strains  and plasmid names | Relevant characteristics | Source of reference |
| *Rhodococus equi* |  |  |  |
|  | ATCC33701 | virulent strain |  |
|  | TKR255 | P*vapA-lacZ* fusion strain of ATCC33701 | (25) |
|  | TKR474 | *virR_ΔHTH_* of TKR255 | (25) |
| *Escherichia coli* |  |  |  |
|  | DH5α | F^+^ *endA1* *glnV44* *thi-1* *recA1* *relA1* *gyrA96* *deoR* *nupG* *purB20* φ80d*lacZ*ΔM15 Δ(*lacZYA-argF*)U169, hsdR17(*r_K_*^–^*m_K_*^+^), λ^+^ |  |
|  | ER2566 | F- λ- fhuA2 [lon] ompT lacZ::T7p07 gal sulA11 Δ(mcrC-mrr)114::IS10 R(mcr-73::miniTn10-TetS)2 R(zgb-210::Tn10)(TetS) endA1 [dcm] |  |
|  | SP850 | 􏰆􏰂λ- *e14*- *relA1 spoT1 cyaA1400*(::*kan*) *thi-1* | *E. coli* Genetic Stock Center, Yale |
| Plasmid |  |  |  |
|  | pINT | pBluescript II SK with aac(3)IV and integrase, Ap^r^, Apr^r^ | (25) |
|  | pTKR528 | pINT with P*_virR_*-*virR* , Ap^r^, Apr^r^ | (25) |
|  | pTKR638 | pINT with P*_virR_*-*virR* (L98E), Ap^r^, Apr^r^ | This study |
|  | pTKR639 | pINT with P*_virR_*-*virR* (S100E), Ap^r^, Apr^r^ | This study |
|  | pTKR640 | pINT with P*_virR_*-*virR* (L101E), Ap^r^, Apr^r^ | This study |
|  | pTXB1 | Expression vector, Ap^r^ | New England  Biolabs |
|  | pTKR752 | pTXB1 with *virR*, Ap^r^ | This study |
|  | pTKR746 | pTXB1with *virR* (L98E) , Ap^r^ | This study |
|  | pGEM T easy | Multi-copy cloning vector, Ap^r^ | Promega |
|  | pTKR837 | pGEM T easy with *virR-icgA* intergenic region, Ap^r^ | This study |
|  | pTKR780 | ΔL of pTKR837, Ap^r^ | This study |
|  | pTKR781 | ΔR of pTKR837 , Ap^r^ | This study |
|  | pTKR801 | RL of pTKR837, Ap^r^ | This study |
|  | pTKR778 | ΔABS of pTKR837, Ap^r^ | This study |
|  | pTKR918 | pUC57 mini with NotI-terminator-PicgA2-t tag-terminator-NotI | This study |
|  | pReporter | pINT with NotI-terminator-PicgA2-t tag-  terminator-NotI | This study |
|  | pTKR919 | Δ-35 of pReporter | This study |
|  | pTKR826 | ΔABS of pReporter | This study |
|  | pTKR943 | RL of pReporter | This study |
|  | pTKR509 | pGEM T easy with PvirR-virR (wild) | This study |
|  | pTKR917 | pGEM T easy with PvirR-virR (L98E) | This study |
|  | pTKR913 | pReporter with P*_virR_*-*virR* (wild) | This study |
|  | pTKR914 | pReporter with P*_virR_*-virR (L98E) | This study |
|  | pBEND2 | Vector for circular permutation analysis, Ap^r^ | (31) |
|  | pTKR810 | pBEND2 with P*icgA* (−80 ~ −30), Ap^r^ | This study |
|  | pT18 | Encodes T18 fragment of CyaA | (33) |
|  | pT25 | Encodes T25 fragment of CyaA | (33) |
|  | pTKR920 | pT18 with *virR* (wild) | This study |
|  | pTKR921 | pT18 with *virR* (L98E) | This study |
|  | pTKR922 | pT18 with *rpoA* | This study |
|  | pTKR923 | pT18 with *zip* | (33) |
|  | pTKR924 | pT25 with *virR* (wild) | This study |
|  | pTKR925 | pT25 with *virR* (L98E) | This study |
|  | pTKR926 | pT25 with *rpoA* | This study |
|  | pTKR927 | pT25 with *zip* | (33) |
