# Supplementary material for: LysR-Type Transcriptional Regulator VirR Responds to Temperature and pH and Directly Activates the Transcription of virS-Containing Operon in Rhodococcus equi
Source: Int J Microbiol. 2025 Jan 3;2025:6618952. doi: 10.1155/ijm/6618952 (PMC11724031; doi:10.1155/ijm/6618952)
Supplement: Supporting Information 2 — Table S2: The primers and oligonucleotides used in this study are listed in Table S2. Their nucleotide sequences and purposes are shown. [file 6618952.f2.docx]

| Table S2. Primers used in this study | | | |
| --- | --- | --- | --- |
| Primer name | Sequence (5′ to 3′) | Purpose | |
| VirR-CTF | GCCGAGACCCACCAAAGAG | Real-time RT PCR | |
| VirR-CTR | CTCCGACATCGTCCAAAGGT | Real-time RT PCR | |
| IcgA-RTF | CAGACACGCGATGTGTGATG | Real-time RT PCR | |
| IcgA-RTR | TCGCAACTCCGATCAGGAAG | Real-time RT PCR | |
| VapH-RTF | GCGACGGTACGATGCTTGT | Real-time RT PCR | |
| VapH-RTR | TGAGTCGATGGCTCGAGAAA | Real-time RT PCR | |
| Orf7-RTF | GGCATGCACTCCCTGAAAAC | Real-time RT PCR | |
| Orf7-RTR | CGATTCGGATTCGAAAGCA | Real-time RT PCR | |
| VirS-RTF | GCGCGCTGCATATATGTGA | Real-time RT PCR | |
| VirS-RTR | CAGGTCGAACTCTCGGGAAGT | Real-time RT PCR | |
| t-tag167F | CACGGCAAGACCATCTACTTC | Real-time RT PCR | |
| t-tag167R | ACGGTCATGAATCGTTCGTT | Real-time RT PCR | |
| gyrB-RTF | CCAGAAGTCAGACAAGAGCAACA | Real-time RT PCR | |
| gyrB-RTR | GACGCTTGCGGACAGCTT | Real-time RT PCR | |
| VirR-NdeF | GGTGGTCATATGAATGTTGACGAACTCCGCTG | Recombinant Protein | |
| VirR-SapR | GGTGGTTGCTCTTCCGCAGGCGCCCTCTTGTCCGCGGT | Recombinant Protein | |
| RT-primer | TCCTACTAGGAC | 5′-RACE | |
| A1 | TCGCAAACAGAATTGACTGTGGTGC | 5′-RACE | |
| A2 | GGTCGCAACTCCGATCAGGAAGAGC | 5′-RACE | |
| S1 | CTACTGCATCGACTGCGGGACTCGC | 5′-RACE | |
| S2 | TGGGTTTTTGTCTCCGACCGAGTGG | 5′-RACE |  |
| P*_virR_*-termF | TGAAAACGGCCCCCGGAGTCTCCTCCGAGGGCCATTTCGCGTAGGACGAGAACCCTCTCC | VirR orf with its promoter |  |
| *virR*-R | CTAGGCGCCCTCTTGTCCGC | VirR orf with its promoter |  |
| VirR L98E-1 | GCACCGTTCGGCTAGGCTTCGAGCACTCCCTTGCGAGTTGGT | Mutagenesis |  |
| VirR L98E-2 | ACCAACTCGCAAGGGAGTGCTCGAAGCCTAGCCGAACGGTGC | Mutagenesis |  |
| VirR S100E-1 | ACCGTTCGGCTAGGCTTCCTGCACGAGCTTGCGAGTTGGTTTGTGCCT | Mutagenesis |  |
| VirR S100E-2 | AGGCACAAACCAACTCGCAAGCTCGTGCAGGAAGCCTAGCCGAACGGT | Mutagenesis |  |
| VirR L101E-1 | ACCGTTCGGCTAGGCTTCCTGCACTCCGAGGCGAGTTGGTTTGTGCCT | Mutagenesis |  |
| VirR L101E-2 | AGGCACAAACCAACTCGCCTCGGAGTGCAGGAAGCCTAGCCGAACGGT | Mutagenesis |  |
| PicgA-F | GGGTGCCGCCCGGAGTGGGT | EMSA |  |
| PicgA-Δ10 | CGGAGTGGGTGGGCCGAGTC | EMSA |  |
| PicgA-Δ20 | GGGCCGAGTCGCGCGCAGAC | EMSA |  |
| PicgA-Δ30 | GCGCGCAGACTTGATGCGGA | EMSA |  |
| PicgA-Δ40 | TTGATGCGGATCACGCATCG | EMSA |  |
| PicgA-Δ50 | TCACGCATCGCAGGTGGTCG | EMSA |  |
| PicgA-Δ60 | CAGGTGGTCGTGAACCGAAT | EMSA |  |
| PicgA-F0 | GTTTCTAGCAAACCGCGGAC | EMSA, DNase I FP |  |
| PicaA-R | AGTGTTCGACGCTAGGAGGG | EMSA, DNase I FP |  |
| PicgA-core1 | CTAGGCAGACTTGATGCGGATCACGCATCGCAGGTGGTCGTGAACCGAATTGGACACTAG | Circular permutation analysis |  |
| PicgA-core2 | TCGACTAGTGTCCAATTCGGTTCACGACCACCTGCGATGCGTGATCCGCATCAAGTCTGC | Circular permutation analysis |  |
| delta-ABS1 | TGCGGATCACGCATCGCAGGTGGACACTAGGCCCAACCCCT | Mutagenesis |  |
| delta-ABS2 | AGGGGTTGGGCCTAGTGTCCACCTGCGATGCGTGATCCGCA | Mutagenesis |  |
| delta-L1 | GCCGAGTCGCGCGCAGACTTGATCACGCATCGCAGGTGGT | Mutagenesis |  |
| delta-L2 | ACCACCTGCGATGCGTGATCAAGTCTGCGCGCGACTCGGC | Mutagenesis |  |
| delta-R1 | CGCAGACTTGATGCGGATCAGCAGGTGGTCGTGAACCGAA | Mutagenesis |  |
| delta-R2 | TTCGGTTCACGACCACCTGCTGATCCGCATCAAGTCTGCG | Mutagenesis |  |
| RL1 | GCGCGCAGACTTGAAGCGGATCACGCTTCGCAGGTGGTCGT | Mutagenesis |  |
| RL2 | ACGACCACCTGCGAAGCGTGATCCGCTTCAAGTCTGCGCGC | Mutagenesis |  |
| delta-35-1 | CAGGTGGTCGTGAACCGAATCTAGGCCCAACCCCTCCTAG | Mutagenesis |  |
| delta-35-2 | CTAGGAGGGGTTGGGCCTAGATTCGGTTCACGACCACCTG | Mutagenesis |  |
| virR-builderR | ACGACGGCCAGTGATTGAAAACGGCCCCCGGAGTC | Bacterial two hybrid system |  |
| virR-builderF | GTCGTATTACGCGATCTAGGCGCCCTCTTGTCCGC | Bacterial two hybrid system |  |
| pT18-1 | CGATACCGTCGACCTCGAGGG | Bacterial two hybrid system |  |
| pT18-2 | ATAAGCTTGATATCGAATTCC | Bacterial two hybrid system |  |
| pT25-1 | CTCTAGAGTCGACCCTGCAGC | Bacterial two hybrid system |  |
| pT25-2 | GATCCCCGGGTACCTAAGTAA | Bacterial two hybrid system |  |
| T18-RpoAF | AGGTCGACGGTATCGATGCTCATTTCTCAGCGACC | Bacterial two hybrid system |  |
| T18-RpoAR | CGATATCAAGCTTATCAGCTGTTCGGTCTCGGCGT | Bacterial two hybrid system |  |
| T25-RpoAF | GGGTCGACTCTAGAGATGCTCATTTCTCAGCGACC | Bacterial two hybrid system |  |
| T25-RpoAR | AGGTACCCGGGGATCCAGCTGTTCGGTCTCGGCGT | Bacterial two hybrid system |  |
| T18-VirRF | AGGTCGACGGTATCGGTGAATGTTGACGAACTCCG | Bacterial two hybrid system |  |
| T18-VirRR | CGATATCAAGCTTATGGCGCCCTCTTGTCCGCGGT | Bacterial two hybrid system |  |
| T25-VirRF | GGGTCGACTCTAGAGGTGAATGTTGACGAACTCCG | Bacterial two hybrid system |  |
| T25-VirRR | AGGTACCCGGGGATCGGCGCCCTCTTGTCCGCGGT | Bacterial two hybrid system |  |

|  |
| --- |
